# A Single-Dose Study of Denosumab in Patients With Various Degrees of Renal Impairment

**DOI:** 10.1002/jbmr.1613

**Published:** 2012-03-28

**Authors:** Geoffrey A Block, Henry G Bone, Liang Fang, Edward Lee, Desmond Padhi

**Affiliations:** 1 Denver Nephrologists, PC, Clinical Research DivisionDenver, CO, USA; 2 Michigan Bone and Mineral ClinicDetroit, MI, USA; 3 Amgen Inc.Thousand Oaks, CA, USA

**Keywords:** CHRONIC KIDNEY DISEASE, BONE, PHARMACOKINETICS, CALCIUM, VITAMIN D

## Abstract

This 16-week study evaluated pharmacokinetics and pharmacodynamics of denosumab in 55 subjects with renal function ranging from normal to dialysis-dependent kidney failure. Participants received a single 60-mg subcutaneous dose of denosumab. Kidney function groups were based on calculations using the Cockcroft-Gault equation and U.S. Food and Drug Administration (FDA) guidance in place when the study was designed. Renal function did not have a significant effect on denosumab pharmacokinetics or pharmacodynamics. These findings suggest denosumab dose adjustment based on glomerular filtration rate is not required. Rapid decreases in serum C-telopeptide in all groups were sustained throughout the study. The most common adverse events were hypocalcemia (15%), pain in extremity (15%), and nausea (11%). Most adverse events were mild to moderate in severity. Calcium and vitamin D supplementation was not initially required by the study protocol, but was added during the trial. No subject who received adequate calcium and vitamin D supplementation became hypocalcemic. Seven subjects had nadir serum calcium concentrations between 7.5 and <8.0 mg/dL (1.9 and <2.0 mmol/L), and 5 subjects (4 with advanced renal disease) had nadir serum calcium <7.5 mg/dL (<1.9 mmol/L). Two subjects (1 symptomatic, 1 asymptomatic) were hospitalized for intravenous calcium gluconate treatment. At the recommended dose, denosumab is a useful therapeutic option for patients with impaired renal function. Supplementation of calcium and vitamin D is strongly recommended when patients initiate denosumab therapy, particularly in patients with reduced renal function. © 2012 American Society for Bone and Mineral Research.

## Introduction

Chronic kidney disease (CKD) may be an independent risk factor for bone loss^1^–^3^ and is common among older adults. In a large study of older adults (mean age, 71.3 years), more than 50% had a glomerular filtration rate (GFR) <60 mL/min/1.73 m^2^, including severe CKD or kidney failure (GFR <30 mL/min/1.73 m^2^) in 3%.^1^ Bisphosphonates are commonly used to reduce bone loss, but they are primarily cleared via renal excretion. Thus, in patients with severe renal impairment, bisphosphonate use has not been extensively investigated and either is not recommended (eg, risedronate, alendronate, ibandronate, tiludronate) or recommended only with precautions and careful monitoring (eg, etidronate, pamidronate).^4^ An August 2011 update to the United States labeling for zoledronic acid in osteoporosis contraindicated its use in patients with GFR <35 mL/min/1.73 m^2^.^5^ However, it is used with dose adjustment in patients with skeletal metastases.

Bisphosphonate treatment, particularly at higher peak concentrations, may also contribute to deterioration of renal function,^4^ and the efficacy of bisphosphonates for bone protection may be limited in patients with severe renal impairment or kidney failure.^6^ Cases of renal failure have been reported among patients who received rapid injections of zoledronic acid for treatment of bone metastases.^7^

Receptor activator of nuclear factor-κB (RANK) ligand (RANKL) is essential in formation, activation, and survival of osteoclasts.^8^–^10^ Denosumab is a fully human monoclonal antibody that has high affinity and specificity for RANKL.^11^ In a placebo-controlled, phase 3 study of 7808 postmenopausal women with osteoporosis, denosumab treatment significantly reduced the risk of vertebral, nonvertebral, and hip fractures.^12^ Furthermore, the benefit was independent of the degree of renal impairment, including Stage 4 CKD.^13^ Other trials demonstrated that subcutaneous denosumab injections every 6 months increased bone mineral density and reduced markers of bone resorption more effectively than once-weekly oral alendronate.^14^, ^15^ In earlier clinical studies, denosumab dose-dependently decreased bone turnover, with rapid onset within 12 hours and sustained effects for up to 6 months after a single dose,^16^ and repeated doses of denosumab every 6 months for up to 6 years increased bone mineral density and decreased bone resorption.^17^–^19^ Denosumab was also reported to be superior to zoledronic acid in preventing skeletal complications of cancer bone metastases.^20^–^22^

The objectives of this study were to evaluate pharmacokinetics, pharmacodynamics, and safety of a single 60-mg subcutaneous dose of denosumab in subjects with various degrees of renal function impairment. In accordance with U.S. Food and Drug Administration (FDA) guidance, which differed somewhat from the most recent National Kidney Foundation Disease Outcomes Quality Initiative (KDOQI) guidelines,^23^ participants were classified into renal function categories ranging from normal to kidney failure requiring dialysis, employing the Cockcroft-Gault equation.^24^

## Patients and Methods

### Study Design

This 16-week, open-label, single-dose, outpatient study was conducted at 12 centers in the United States. Subjects were screened for eligibility up to 21 days before study treatment, and baseline evaluations were conducted on day –1 before study treatment. In accordance with regulatory guidelines,^25^ subjects were enrolled in one of five renal function groups based on GFR at baseline by Cockcroft-Gault estimation: normal renal function (GFR >80 mL/min/1.73 m^2^); mild CKD (GFR 50–80 mL/min/1.73 m^2^); moderate CKD (GFR 30–49 mL/min/1.73 m^2^); severe CKD (GFR <30 mL/min/1.73 m^2^); or kidney failure requiring hemodialysis.

Eligible subjects received a single 60-mg subcutaneous dose of denosumab (Prolia; Amgen, Inc., Thousand Oaks, CA, USA) on day 1. Concomitant medications could include acetaminophen, nonsteroidal anti-inflammatory drugs, vitamins, hormonal contraceptives, topical medications, and treatments for the subject's renal disease or associated conditions.

Subjects returned for follow-up visits on days 2, 3, 6, 8, 11, 15, 22, 29, 43, 57, 85, and 113. At each visit, adverse events were recorded and blood samples were drawn for measurement of serum denosumab concentrations for pharmacokinetic analysis and serum C-telopeptide (sCTX1) for pharmacodynamic analysis. Serum concentrations of denosumab were measured at a central laboratory (PPD Development, Richmond, VA, USA) by a validated conventional sandwich enzyme-linked immunosorbent assay (ELISA). The lower limit of quantification was 0.8 ng/mL (800 ng/L).

Blood samples for analysis of anti-denosumab antibodies were obtained predose on day 1 and on day 113 (or at early termination), and measured at a central laboratory (Amgen and PPD Development). An electrochemiluminescent bridging immunoassay was used to test for binding antibodies to denosumab.

### Study participants

Key inclusion criteria were age ≥18 years, normal laboratory tests at screening and baseline (other than tests expected to be outside the normal range in subjects with CKD), clinically acceptable physical examination and electrocardiograph (ECG) results at screening, and use of an effective barrier method of contraception during the study. Key exclusion criteria were known sensitivity to any study treatment, any unstable medical condition, human immunodeficiency virus (HIV), hepatitis B (permitted in the kidney failure group), hepatitis C, history of malignancy, active infection, pregnancy, lactation/nursing, known alcohol or drug abuse, albumin-adjusted serum calcium <8.5 mg/dL (2.1 mmol/L) at screening, and 25-hydroxyvitamin D (25-OH D; calcifediol) <30 ng/mL (<75 nmol/L) at screening.

Among the first 24 subjects enrolled, hypocalcemia was seen in the severe CKD group (see Results). The study protocol was therefore amended to include requirements for daily supplementation of calcium (up to 1000 mg) and vitamin D (up to 800 IU) in all subjects with CKD; and to exclude subjects from study who had severe CKD and 1,25-dihydroxyvitamin D (1,25-[OH]_2_D; calcitriol) <30 pg/mL, severe CKD and intact parathyroid hormone (iPTH) ≥110 pg/mL (≥110 ng/L), or kidney failure and iPTH ≥300 pg/mL (≥300 ng/L). After the amendments, 3 additional subjects each in the mild CKD and moderate CKD groups were enrolled and safety data through day 15 were reviewed before enrollment in the severe CKD group was recommenced.

An Institutional Review Board approved the study for each site. The study was conducted in accordance with the principles of the Declaration of Helsinki. Investigators obtained written informed consent from each subject prior to participation.

### Statistical analysis

Primary endpoints were pharmacokinetic parameters, including the area under the serum-concentration time curve (AUC_0–113 days_) and maximum concentration (C_max_). The pharmacokinetic analyses included all subjects for whom the pharmacokinetic parameters could be derived. One subject in the normal group was lost to follow-up after day 14 and was excluded from all other pharmacokinetic analyses. Means for each renal function group were calculated. A nonparametric test for trend analysis was used to assess the effect of decreasing renal function on denosumab disposition. Regression analysis was used to model the relationship between the values of the pharmacokinetic parameters and GFR.

Secondary endpoints included subject incidences of adverse events, clinically significant changes in vital signs, physical examinations, clinical laboratory tests, ECGs, and incidence of anti-denosumab antibodies. Subject incidences of albumin-adjusted serum calcium concentrations <8.0 mg/dL (<2.0 mmol/L), albumin-adjusted serum calcium concentration <7.5 mg/dL (<1.9 mmol/L), serious adverse events of hypocalcemia, and symptomatic adverse events of hypocalcemia were evaluated by renal function group. A post hoc multivariate analysis was conducted to evaluate the association between mean decrease from baseline to nadir serum calcium concentration and baseline characteristics (age, race, sex, CKD stage, serum calcium, serum phosphorus, iPTH, 25-OHD, and 1,25-(OH)_2_D). Proportions were calculated for subjects with at least a 50% reduction in GFR using the Modification of Diet in Renal Disease (MDRD) equation.^23^

Change in sCTX1 concentrations was an exploratory pharmacodynamic endpoint; sCTX1 values below the limit of quantification were set to zero. The Spearman correlation coefficient was calculated for maximum absolute decrease in sCTX1 and (1) baseline iPTH and (2) baseline alkaline phosphatase.

The planned sample size of 46 subjects (12 with normal renal function, 10 each with mild and moderate CKD, 7 with severe CKD, and 7 with kidney failure) was based on practical considerations. After the study protocol was amended to include calcium and vitamin D supplementation, enrollment was increased to include 13 subjects each in the mild CKD and moderate CKD groups, and 8 to 9 subjects each in the severe CKD and kidney failure groups.

## Results

### Baseline characteristics

Table 1 summarizes baseline characteristics by renal function group. Fifty-five subjects were enrolled, including 12, 13, 13, 9, and 8 subjects in the normal, mild CKD, moderate CKD, severe CKD, and kidney failure (dialysis) groups, respectively, based on estimated GFR. Similar proportions of women (51%) and men (49%) participated. Most subjects (69%) were white. Mean ± SD age at baseline was 64 ± 15 years. Baseline subject characteristics were generally well balanced across renal function groups, but more subjects were Black/African American in the kidney failure group (63%) than in other groups (0% to 38%).

**Table 1:** Baseline Demographics and Laboratory Values

	Renal function group					
	
	Normal (*n* = 12)	Mild CKD (*n* = 13)	Moderate CKD (*n* = 13)	Severe CKD (*n* = 9)	Kidney failure (*n* = 8)	Total (*n* = 55)
Female	7 (58)	8 (62)	6 (46)	5 (56)	2 (25)	28 (51)
Race/ethnicity						
White/Caucasian	10 (83)	7 (54)	11 (85)	8 (89)	2 (25)	38 (69)
Black/African American	0 (0)	5 (38)	0 (0)	1 (11)	5 (63)	11 (20)
Hispanic/Latino	2 (17)	1 (8)	2 (15)	0 (0)	1 (13)	6 (11)
Age, years	54 ± 9	63 ± 10	72 ± 11	66 ± 24	67 ± 15	64 ± 15
iPTH (pg/mL)^a^						
Before amendment^b^	24.8 ± 0.4 (*n* = 2)	– (*n* = 0)	71.1 ± 38.2 (*n* = 7)	164.0 ± 75.0 (*n* = 2)	– (*n* = 0)	79.6 ± 59.3 (*n* = 11)
After amendment^b^	32.3 ± 18.3 (*n* = 7)	64.2 ± 34.5 (*n* = 6)	53.2 ± 31.6 (*n* = 6)	82.6 ± 48.6 (*n* = 7)	158.2 ± 125.1 (*n* = 5)	74.2 ± 68.6 (*n* = 31)
Calcium (mg/dL)^a^^,^^c^						
Before amendment^b^	9.6 ± 0.5 (*n* =5)	10.1 ± 0.9 (*n* =7)	9.4 ± 0.5 (*n* =7)	9.1 ± 0.5 (*n* =2)	9.4 ± 0.5 (*n* =3)	9.6 ± 0.7 (*n* =24)
After amendment^b^	9.3 ± 0.3 (*n* = 7)	9.3 ± 0.2 (*n* = 6)	9.6 ± 0.2 (*n* = 6)	9.6 ± 0.6 (*n* = 7)	9.1 ± 0.4 (*n* = 5)	9.4 ± 0.4 (*n* = 31)
25-OHD (nmol/L)						
Before amendment^b^	99.1 ± 15.2 (*n* =2)	– (*n* =0)	118.4 ± 31.5 (*n* =7)	141.0 ± 54.7 (*n* =2)	– (*n* = 0)	119.0 ± 33.1 (*n* =11)
After amendment^b^	101.9 ± 52.5 (*n* = 7)	106.3 ± 29.6 (*n* = 6)	99.2 ± 66.1 (*n* = 6)	76.9 ± 54.9 (*n* = 7)	39.2 ± 40.4 (*n* = 5)	86.5 ± 53.0 (*n* = 31)
1,25-(OH)_2_D (pmol/L)						
Before amendment^b^	– (*n* = 0)	– (*n* = 0)	– (*n* = 0)	– (*n* = 0)	– (*n* = 0)	– (*n* = 0)
After amendment^b^	113.8 ± 32.2 (*n* = 7)	137.2 ± 91.4 (*n* = 6)	93.0 ± 31.7 (*n* = 6)	125.9 ± 58.9 (*n* = 7)	46.7 ± 23.5 (*n* = 5)	106.2 ± 58.8 (*n* = 31)

Values are *n* (%) or mean ± SD.

CKD = chronic kidney disease; iPTH = intact parathyroid hormone; 25-OHD = 25-hydroxyvitamin D; 1,25-(OH)_2_D = 1,25-dihydroxyvitamin D.

a SI unit conversions: calcium, 4.0 mg/dL = 1.0 mmol/L; iPTH, 1 pg/mL = 1 ng/L.

b Study amendment added calcium and vitamin D supplementation, and excluded subjects with low 1,25-(OH)_2_D or high iPTH (see Study Participants).

c Albumin-adjusted serum calcium.

Baseline iPTH, 25-OHD, and 1,25-(OH)_2_D assessments were not initially required; therefore, only a subset of subjects had baseline data for these parameters. Baseline iPTH concentrations generally were higher in subjects with more severe renal impairment. Baseline albumin-adjusted serum calcium concentrations were similar across renal function groups. Baseline 25-OHD and 1,25-(OH)_2_D concentrations were variable across and within groups.

Of the 55 subjects enrolled, 95% completed the study. Reasons for early study withdrawal included 1 subject (normal) who withdrew consent due to a family emergency, 1 subject (mild CKD) lost to follow-up, and 1 subject (severe CKD) who asked to be withdrawn due to a busy personal schedule.

### Pharmacokinetics

**Fig. 1:**
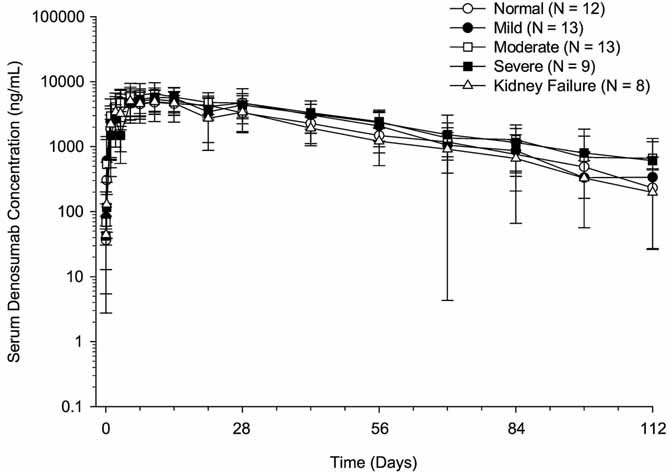
Mean ± SD serum denosumab concentration-time profiles after subcutaneous administration of denosumab.

Mean serum denosumab concentration-time profiles after subcutaneous administration of denosumab 60 mg were similar between renal function groups (Fig. 1). Table 2 summarizes pharmacokinetic parameter estimates by renal function group. Median time to maximum denosumab concentration (T_max_) was the same in each group (10 days). Mean values for area under the denosumab concentration curve from the start of treatment until day 113 (AUC_0–113 days_) and C_max_ did not differ significantly between renal function groups. Linear regression analyses did not demonstrate a significant relationship between baseline GFR and AUC_0–113 days_ (*p* = 0.173) or between GFR and C_max_ (*p* = 0.334). Nonparametric analysis (Jonckheere-Terpstra trend test) did not demonstrate a significant association between renal function group and either AUC_0–113 days_ (*p* = 0.595) or C_max_ (*p* = 0.511).

**Table 2:** Serum Denosumab Pharmacokinetic Parameter Estimates for a Single Subcutaneous Dose of Denosumab

	Renal function group					*p*	
		
	Normal (*n* = 11)	Mild CKD (*n* = 13)	Moderate CKD (*n* = 13)	Severe CKD (*n* = 9)	Kidney failure (*n* = 8)	Linear regression^a^	Jonckheere-Terpstra^b^
AUC_0–113 days_ (µg^*^day/mL)	217 ± 76	266 ± 143	322 ± 154	295 ± 120	208 ± 107	0.173	0.595
C_max_ (ng/mL)	5160 ± 1770	6200 ± 2880	7040 ± 3060	6020 ± 2320	5370 ± 2590	0.334	0.511
T_max_ (day)	10 (3–14)	10 (2–28)	10 (3–28)	10 (7–14)	10 (5–21)		–

Values are mean ± SD or median (range).

CKD = chronic kidney disease; AUC_0–113 days_ = area under the serum-concentration time curve; C_max_= maximum concentration; T_max_ = time to maximum denosumab concentration; GFR = glomerular filtration rate.

a Regression analysis of the relationship between GFR and pharmacokinetic parameters.

b Obtained from nonparametric Jonckheere-Terpstra trend test.

### Pharmacodynamics

**Fig. 2:**
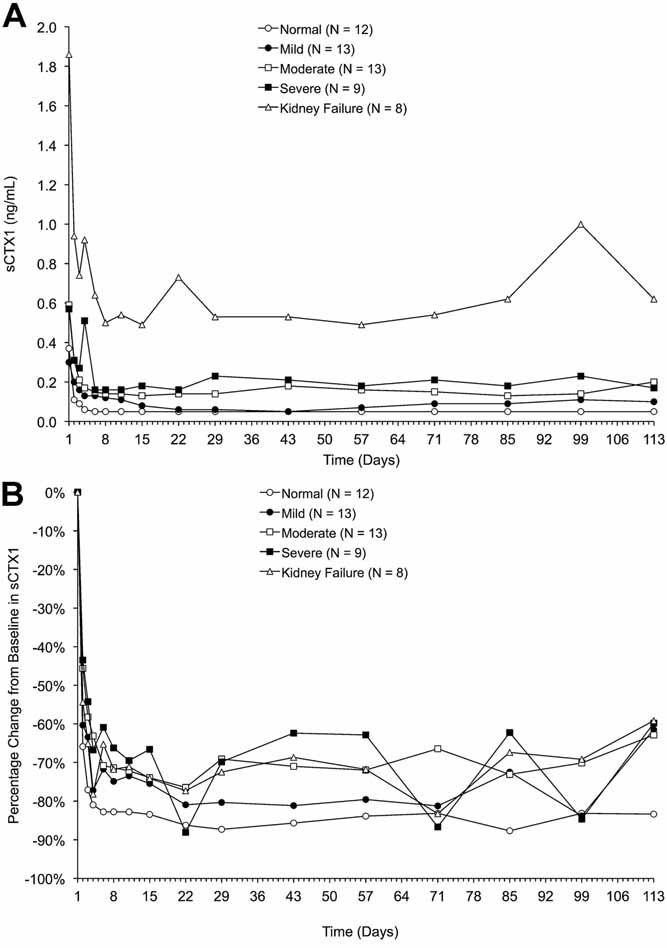
Serum C-telopeptide (sCTX1) concentrations after a single subcutaneous injection of denosumab. (*A*) Median values for sCTX1 by renal function group. (*B*) Median percent change from baseline in sCTX1. SI unit conversion: sCTX1, 1.0 ng/mL = 1000 ng/L.

In each renal function group, denosumab treatment resulted in rapid decreases from baseline in sCTX1 concentrations that were sustained from the first observation at day 2 to the end of study (Fig. 2*A*). As expected, the kidney failure group had higher sCTX1 concentrations than other groups at baseline and throughout study, but relative decreases from baseline in sCTX1 were generally similar (65%–85%) across renal function groups (Fig. 2*B*). The normal group tended to have the lowest absolute sCTX1 concentrations after denosumab administration and the greatest relative reductions in sCTX1 from baseline. Spearman correlation coefficients for maximum decrease in sCTX1 were 0.449 for baseline iPTH (*p* = 0.003) and 0.181 for baseline alkaline phosphatase (*p* = 0.186).

### Adverse events

Most subjects (80%) had at least one adverse event (Table 3), including 50%, 77%, 92%, 89%, and 100% of subjects in the normal, mild CKD, moderate CKD, severe CKD, and kidney failure groups, respectively. Most adverse events were mild to moderate in severity. Clinical adverse events reported by at least 10% of subjects overall were pain in extremity (15%) and nausea (11%). Hypocalcemia was observed in 15% of patients, only 1 of whom was symptomatic (see the next paragraph). Adverse events that investigators considered related to study treatment or procedures in at least 5% of subjects were hypocalcemia (13%), vessel puncture site hematoma (9%), secondary hyperparathyroidism (5%), and muscle spasms (5%). No subject withdrew because of an adverse event, and no subject died during the study.

**Table 3:** Adverse Events Reported in ≥5% of Subjects, by Descending Order of Frequency Overall

	Normal (*n* = 12)	Mild CKD (*n* = 13)	Moderate CKD (*n* = 13)	Severe CKD (*n* = 9)	Kidney failure (*n* = 8)	Total (*n* = 55)
Any adverse event	6 (50)	10 (77)	12 (92)	8 (89)	8 (100)	44 (80)
Hypocalcemia	0 (0)	1 (8)	3 (23)	2 (22)	2 (25)	8 (15)
Pain in extremity	0 (0)	3 (23)	2 (15)	2 (22)	1 (13)	8 (15)
Nausea	0 (0)	2 (15)	1 (8)	0 (0)	3 (38)	6 (11)
Vessel puncture site hematoma	0 (0)	1 (8)	3 (23)	1 (11)	0 (0)	5 (9)
Arthralgia	0 (0)	2 (15)	1 (8)	1 (11)	0 (0)	4 (7)
Headache	0 (0)	2 (15)	1 (8)	1 (11)	0 (0)	4 (7)
Secondary hyperparathyroidism	0 (0)	0 (0)	2 (15)	0 (0)	2 (25)	4 (7)
Muscle spasms	0 (0)	0 (0)	2 (15)	2 (22)	0 (0)	4 (7)
Pharyngolaryngeal pain	1 (8)	0 (0)	0 (0)	2 (22)	1 (13)	4 (7)
Urinary tract infection	0 (0)	1 (8)	2 (15)	1 (11)	0 (0)	4 (7)
Back pain	0 (0)	1 (8)	2 (15)	0 (0)	0 (0)	3 (5)
Contusion	0 (0)	1 (8)	2 (15)	0 (0)	0 (0)	3 (5)
Edema peripheral	1 (8)	2 (15)	0 (0)	0 (0)	0 (0)	3 (5)
Upper respiratory tract infection	1 (8)	1 (8)	1 (8)	0 (0)	0 (0)	3 (5)
Vomiting	0 (0)	2 (15)	0 (0)	0 (0)	1 (13)	3 (5)

Values are *n* (%).

CKD = chronic kidney disease.

Two subjects (4%) each experienced an adverse event of hypocalcemia that was classified as serious due to hospital treatment (Table 4); both were considered related to denosumab. Both subjects had severe CKD and were enrolled before the protocol required baseline iPTH assessment or supplementation of calcium and vitamin D. Both subjects were hospitalized for intravenous calcium gluconate treatment, and calcium concentrations improved in both subjects after this treatment. One subject was symptomatic (perioral numbness with numbness and tingling of both feet) and the other subject was asymptomatic. No other serious, treatment-related adverse events were reported.

**Table 4:** Subjects With Low Serum Calcium Level or a Serious or Symptomatic Hypocalcemia Adverse Event

	Renal function group					
	
	Normal (*n* = 12)	Mild CKD (*n* = 13)	Moderate CKD (*n* = 13)	Severe CKD (*n* = 9)	Kidney failure (*n* = 8)	Total (*n* = 55)
Calcium <8.0 mg/dL^a^						
Calcium 7.5 to <8.0 mg/dL	0 (0)	1 (8)	2 (15)	1 (11)	3 (38)	7 (13)
Calcium <7.5 mg/dL	0 (0)	1 (8)	0 (0)	2 (22)	2 (25)	5 (9)
Hypocalcemia adverse events						
Serious adverse event	0 (0)	0 (0)	0 (0)	2 (22)	0 (0)	2 (4)
Symptomatic adverse event	0 (0)	0 (0)	0 (0)	1 (11)	0 (0)	1 (2)

Values are *n* (%).

CKD = chronic kidney disease.

a SI unit conversion: calcium, 4.0 mg/dL = 1.0 mmol/L.

### Clinical laboratory evaluations

All subjects tested negative for binding anti-denosumab antibodies. No clinically relevant changes occurred in serum chemistry, hematology, or urinalyses except decreases from baseline in serum concentrations of calcium, phosphorus, and alkaline phosphatase. No adverse events of hypophosphatemia were reported, and concentrations of alkaline phosphatase remained above the lower limit of normal, except for transient decreases in 3 subjects.

**Fig. 3:**
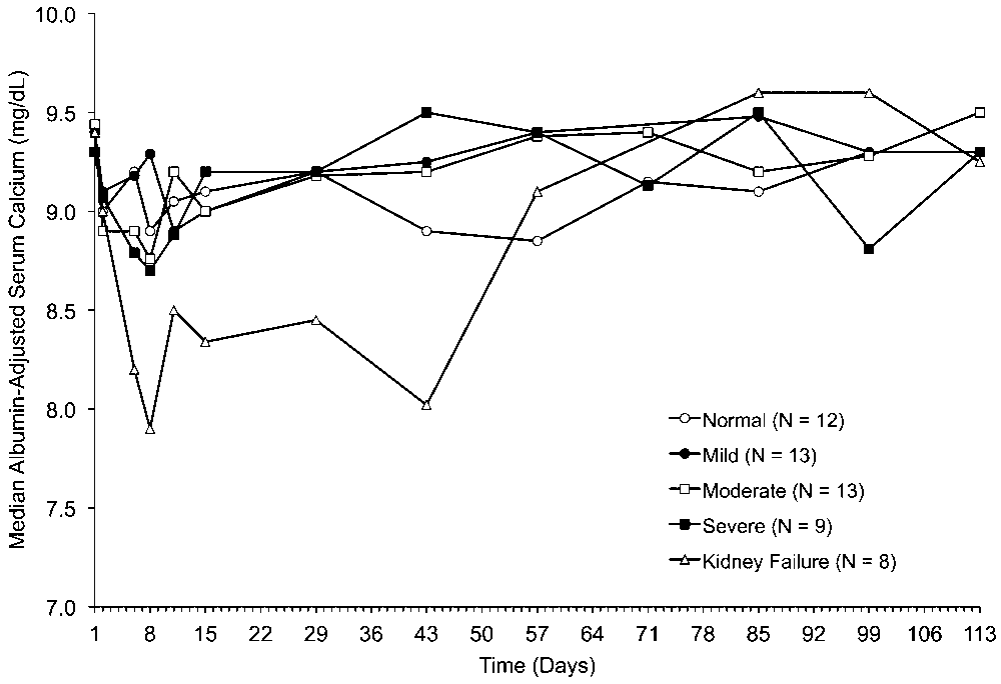
Median albumin-adjusted serum calcium concentrations after a single subcutaneous injection of denosumab. SI unit conversion: calcium, 4.0 mg/dL = 1.0 mmol/L.

Median values for serum calcium are shown in Figure 3. A total of 7 subjects had nadir serum calcium concentrations between 7.5 and <8.0 mg/dL (1.9 to <2.0 mmol/L), and 5 subjects had nadir concentrations <7.5 mg/dL (<1.9 mmol/L) (Table 4); serum calcium returned to >8.0 mg/dL (>2.0 mmol/L) by the end of study for each subject. Only the 1 subject previously mentioned was symptomatic. None of the 5 subjects with nadir serum calcium concentrations <7.5 mg/dL (<1.9 mmol/L) had taken adequate supplementation of calcium and vitamin D. Three of the subjects (1 mild CKD, 2 severe CKD) were enrolled before calcium and vitamin D supplementation was required. Two subjects (both kidney failure) were enrolled after supplementation was required, but were nonadherent to calcium supplementation and had prior histories of intermittent hypocalcemia.

In covariate analysis (Table 5), a nonsignificant trend toward an association between decreased serum calcium and severity of renal impairment was observed. However, the analysis was limited to 31 subjects with data for each factor evaluated and did not have sufficient power to show a statistically significant association between decrease in serum calcium concentration from baseline and the factors: baseline age, race, sex, CKD stage, calcium, phosphorus, iPTH, 25-OHD, or 1,25-(OH)_2_D. There were too few subjects with hypocalcemia to conduct a multivariate analysis of correlations between baseline factors and hypocalcemia.

**Table 5:** Multivariate Analysis of Percentage Change in Albumin-Adjusted Serum Calcium From Baseline to Nadir by Baseline Characteristics (*n* = 31)

Baseline parameter	Parameter estimate	Standard error	*p*
Age	0.08592	0.06111	0.174
Race	1.02376	1.33624	0.452
Sex	−1.60003	1.96521	0.425
Stage of CKD	−1.66602	0.89696	0.077
Albumin-adjusted serum calcium	−12.12495	10.69505	0.270
Phosphorus	−4.64514	3.73841	0.228
iPTH	0.07561	0.20322	0.714
25-OHD	0.02554	0.02151	0.248
1,25-(OH)_2_D	0.02005	0.02189	0.370

CKD = chronic kidney disease; iPTH = intact parathyroid hormone; 25-OHD = 25-hydroxyvitamin D; 1,25-(OH)_2_D = 1,25-dihydroxyvitamin D.

### Renal function

One subject with severe CKD had a >50% decrease in GFR on study (estimated GFR decrease from 14 to 6 mL/min/1.73 m^2^) and was started on renal replacement therapy at day 55. No other subject experienced a clinically significant change in GFR.

## Discussion

In this study, renal function impairment had no significant effect on the pharmacokinetic profile of denosumab in linear regression or nonparametric analyses. Mean values for AUC_0–113 days_ and C_max_ differed by <50% for each CKD group compared with the normal renal function group and were small compared with the large range in exposures within the normal renal function group (approximately threefold for both AUC_0–113 days_ and C_max_).

Higher baseline concentrations of sCTX1 in the kidney failure group and lower concentrations in the normal group, as compared with the other CKD groups in this study, were consistent with previous evidence that sCTX1 levels are higher in patients with impaired renal function,^26^ due to both higher bone turnover and reduced clearance of sCTX1. Relative reductions in sCTX1 were similar across renal function groups, which suggest renal impairment did not influence the pharmacodynamic effects of denosumab on bone turnover.

In preclinical studies, denosumab was not toxic in human renal cells at concentrations up to 100 µmol/L (1 × 10^7^ ng/mL, well above the mean C_max_ values of 5160 to 7040 ng/mL reported in this study), whereas bisphosphonates were nephrotoxic at concentrations above 10 µmol/L.^27^ Although the present study was not designed to estimate the effect of denosumab on kidney function, changes in renal function were consistent with those expected during 16 weeks of follow-up.

A single dose of denosumab 60 mg was generally well tolerated. No subject withdrew from the study due to an adverse event. Most adverse events were mild to moderate in severity. Hypocalcemia, the only treatment-related serious adverse event during the study, required treatment in 2 subjects with severe CKD and was symptomatic in 1 of these subjects. Five subjects had transient decreases in albumin-adjusted serum calcium concentration to <7.5 mg/dL (<1.9 mmol/L), including 3 who were enrolled before a protocol amendment required calcium and vitamin D supplementation and 2 (both with kidney failure) who were nonadherent to calcium and vitamin D supplementation requirements and had prior histories of intermittent hypocalcemia.

In randomized, controlled studies that included more than 900 postmenopausal women who received denosumab for as long as 6 years,^14^, ^17^–^19^ less than 1% of subjects had transient decreases in serum calcium below the reference range and there were no reports of symptomatic hypocalcemia. While the earlier studies excluded women with GFR <35 mL/min/1.73 m^2^ (ie, severe CKD or kidney failure), the pivotal, placebo-controlled phase 3 study of denosumab included 7808 postmenopausal women with no restrictions on renal function; 3 subjects in the placebo group and no subject in the denosumab group had an adverse event of hypocalcemia.^12^ A recent analysis of the women in that study did not find any significant interactions between the severity of CKD and efficacy, safety, or serum calcium, and the efficacy of denosumab was similar in study participants with impaired renal function.^13^

Secondary hyperparathyroidism is a hallmark of chronic renal failure. It results in accelerated bone resorption and formation. The increased amount of new matrix formed may be mineralized in large part by calcium released from bone undergoing resorption. In a manner similar to the “hungry bones” syndrome after parathyroidectomy,^28^ in patients with high-turnover bone disease, following the first dose of denosumab, the endogenous supply of calcium from bone is rapidly reduced but deposition of mineral (calcium and phosphate) into new matrix remains increased. In the time required for recently formed osteoid to adequately mineralize, hypocalcemia may result, unless calcium is appropriately supplemented. Initially, the design of this study did not include calcium supplementation. After modification of the study design, no subject who received adequate calcium supplementation developed hypocalcemia.

Although patients with relatively severe hyperparathyroidism (severe CKD and iPTH ≥110 pg/mL, or kidney failure and iPTH ≥300 pg/mL) were excluded from the study after the study protocol was amended, baseline iPTH levels were still higher among subjects with greater renal impairment in this study. Adequate management of PTH levels is also important in these patients.

In conclusion, results from this study indicate that renal function impairment does not significantly affect the pharmacokinetics and pharmacodynamics of denosumab, and, therefore, dose adjustments are not required for denosumab administration in these patients. As expected with antiresorptive therapy (and consistent with other denosumab studies^14^, ^17^) transient decreases in serum calcium concentration were observed after denosumab administration. The potential for hypocalcemia in subjects with severe CKD and kidney failure appeared greater compared with subjects with mild or moderate CKD and subjects with normal renal function. In patients with impaired renal function that receive denosumab, particularly those with severe kidney disease (defined herein as a GFR <30 mL/min/1.73 m^2^), it is important to provide adequate supplementation of calcium and vitamin D and to adequately manage secondary hyperparathyroidism.
